# Intrafractional Diaphragm Variations During Breath-Hold Stereotactic Body Radiotherapy for a Liver Tumor Based on Real-Time Registration Between Kilovoltage Projection Streaming and Digitally Reconstructed Radiograph Images: A Case Report

**DOI:** 10.7759/cureus.48188

**Published:** 2023-11-02

**Authors:** Atsuto Katano, Yuki Nozawa, Masanari Minamitani, Shingo Ohira, Keiichi Nakagawa

**Affiliations:** 1 Radiology, The University of Tokyo Hospital, Tokyo, JPN; 2 Comprehensive Radiation Oncology, The University of Tokyo, Tokyo, JPN

**Keywords:** volumetric modulated arc therapy, breath-hold, digitally reconstructed radiograph, diaphragm position, liver cancer, stereotactic body radiotherapy

## Abstract

In liver stereotactic body radiotherapy (SBRT), precise image guidance is paramount, serving as the foundation of this treatment approach. The accuracy of SBRT in liver cancer treatment heavily relies on meticulous imaging techniques. The diaphragm, situated adjacent to the liver, is a crucial anatomical structure susceptible to positional and motion variations, which can potentially impact the accuracy of liver tumor targeting. This study explores the application of real-time kilovoltage projection streaming images (KVPSI) in comparison to digitally reconstructed radiography (DRR) for assessing diaphragm position deviations during breath-hold liver tumor SBRT. A 76-year-old male diagnosed with cholangiocarcinoma underwent breath-hold SBRT using split arc volumetric modulated arc therapy (VMAT), where a full arc was split into six sub-arcs, each spanning 60 degrees. The diaphragm dome positions were continuously monitored through KVPSI during treatment. The intrafractional position deviations of the diaphragm were calculated and analyzed for each split arc. The case report revealed a mean diaphragm dome deviation of 0.47 mm (standard deviation: 4.47 mm) in the entire arc. This pioneering study showcases the feasibility of intrafractional diaphragm position variation assessment using real-time KVPSI during the breath-hold liver tumor VMAT-SBRT. Integrating real-time imaging techniques enhances our comprehension of the intra-breath-hold variations, thereby guiding adaptive treatment strategies and potentially improving treatment outcomes. Clinical validation through further research is essential.

## Introduction

Stereotactic body radiotherapy (SBRT) has emerged as a promising non-invasive treatment option for liver tumors, including hepatocellular carcinoma (HCC) and cholangiocarcinoma [1]. This innovative approach offers high local control rates and is associated with low rates of severe toxicity, thus making it a viable choice for liver tumor patients [2]. SBRT works by delivering precise and focused radiation directly to tumors while minimizing damage to healthy liver tissues. Recently, SBRT is often combined with complementary systemic treatments, such as chemotherapy, targeted therapies, nanoparticles, and immunotherapy, to further enhance its effectiveness for HCC [3]. This comprehensive approach not only addresses the challenges posed by advanced-stage HCC but also showcases the potential of SBRT in improving treatment outcomes and quality of life for the patients.

In liver SBRT, image guidance plays a pivotal role and is considered the cornerstone of this treatment modality [4]. The breath-hold technique significantly reduces radiation exposure to the bowel and normal liver tissues compared to the free-breathing method [5]. The precision and effectiveness of SBRT in liver cancer treatment rely heavily on the accuracy of image guidance techniques. The diaphragm, an anatomical structure adjacent to the liver, can exhibit variations in position and motion, potentially affecting the accuracy of liver tumor targeting. According to the European Society for Radiotherapy and Oncology - Advisory Committee for Radiation Oncology Practice (ESTRO-ACROP) guideline about breath-hold techniques in radiotherapy, the diaphragm dome is frequently chosen for a surrogate structure of liver tumors [6]. Traditionally, the diaphragm position has been assessed using static imaging techniques, such as digitally reconstructed radiograph (DRR) images calculated from planning computed tomography (CT) [7]. However, these methods may not capture intra-breath-hold variations in the diaphragm position, which can occur due to physiological events, such as respiration. Real-time respiratory motion management has been widely adopted as a promising approach to monitoring organ motions during treatment delivery [8]. The American Association of Physicists in Medicine Task Group 76 report suggests employing active motion management in cases where respiratory motion surpasses a 5 mm amplitude [9]. This is particularly advantageous in the context of SBRT, where the achievement of optimal normal organ sparing is frequently required for target dose intensification.

In this case report, we aim to assess the intra-breath-hold variations of the diaphragm position during SBRT for liver tumors by comparing real-time kilovoltage projection streaming images (KVPSI) with DRR images calculated from the planning CT. This innovative technique enables us to assess the dynamic shifts in diaphragm position within a single breath-hold, thereby offering valuable insights into the possible sources of uncertainty in tumor targeting. Detailed information on these technologies was outlined in our previous report [10]. Understanding the intra-breath-hold variations of the diaphragm position is crucial for optimizing treatment planning and delivery strategies in SBRT for liver tumors. By characterizing these variations, we can identify potential areas of improvement in target localization and implement appropriate strategies to mitigate the impact of diaphragm motion on treatment outcomes.

## Case presentation

A 76-year-old male patient diagnosed with cholangiocarcinoma was referred to our department for SBRT. Magnetic resonance imaging (MRI) revealed a thickened lesion within the lumen of the hilar bile duct, accompanied by proximal wall thickening involving the left hepatic duct, anterior segmental bile duct, and posterior segmental bile duct. Surgical resection was deemed unsuitable for the patient due to his inability to undergo percutaneous transhepatic portal vein embolization, attributed to elevated portal vein pressure. The patient presented with a serum bilirubin level of 1.7 mg/dL, albumin concentration of 3.0 g/dL, and a prothrombin time international normalized ratio of 1.11. There were no signs of ascites or encephalopathy. Based on the assessment, the patient was classified as having Child-Pugh Class A liver cirrhosis.

**Figure 1:**
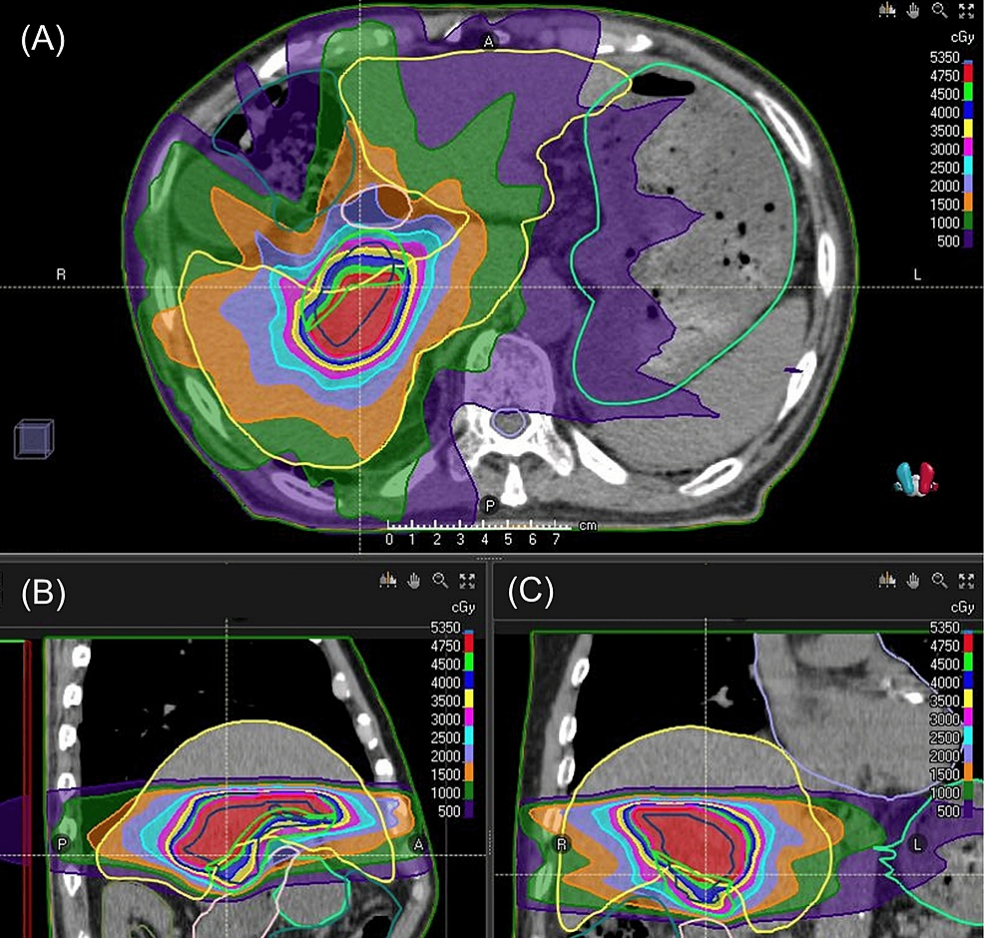
Treatment planning of stereotactic radiotherapy Treatment planning of stereotactic radiotherapy for the liver tumor represented on the axial (A), sagittal (B), and coronal planes (C).

For SBRT treatment planning, an abdominal breath-hold CT scan was performed in the supine position. The gross tumor volume (GTV) was contoured on the breath-hold phase, and a 5 mm isotropic margin was added to generate the planning target volume (PTV). The flattening filter-free beam (FFF) of 6 megavoltage X-ray was used for the dose delivery on an Elekta Versa HD linear accelerator (Elekta, Stockholm, Sweden). A volumetric modulated arc therapy (VMAT) plan was created using RayStation (RaySearch Laboratories, Stockholm, Sweden) treatment planning system, with the aim of delivering 50 Gy in 10 fractions to 50% of the PTV volume (Figure 1).

A single full-arc VMAT was employed for this treatment with a clockwise rotation from -179 degrees to 179 degrees. The arc was split into six partial arcs, each spanning 60 degrees. To facilitate the breath-hold, the beam-on time for each partial arc was limited to less than 20 seconds. This technique, known as split VMAT, was previously proposed to ensure precise delivery of the prescribed dose while accommodating the patient's breath-holding capabilities [11].

On the day of dose delivery, an initial three-dimensional image-matching process was performed between the breath-hold planning CT images and the pre-treatment cone-beam computed tomography (CBCT). During the VMAT delivery, the position of the diaphragm dome was monitored by KVPSI, which was continuously compared to that on a DRR image having the same projection angle. KVPSI provided real-time images of the diaphragm dome position during treatment, while DRR images were calculated every one degree of the gantry rotation by referring to the planning CT volume prior to the VMAT delivery. To assess intra-fractional variations in the diaphragm dome position, a comparison was made every 180 milliseconds between the positions observed in KVPSI and DRR as the reference position from the breath-hold planning CT.

Real-time comparison of KVPSI and DRR images allowed for a continuous monitoring of the diaphragm dome position during the VMAT treatment. Within a span of 180 ms, the projection image was displaced every 1 mm in the superior-inferior direction, and cross correlations between each of the displaced projection images and the DRR image were calculated (Video 1).

**Video 1:** Comparison of diaphragm positions depicted in digitally reconstructed radiography and kilovoltage projection streaming images. This video compares diaphragm positions between the digitally reconstructed radiography (DRR) (left) and kV projection streaming images (right) acquired every 180 ms during six-times split-VMAT deliveries with a clockwise gantry rotation from -179 to 179 degrees, each spanning 60 degrees with a duration of approximately 20 seconds.

**Figure 2:**
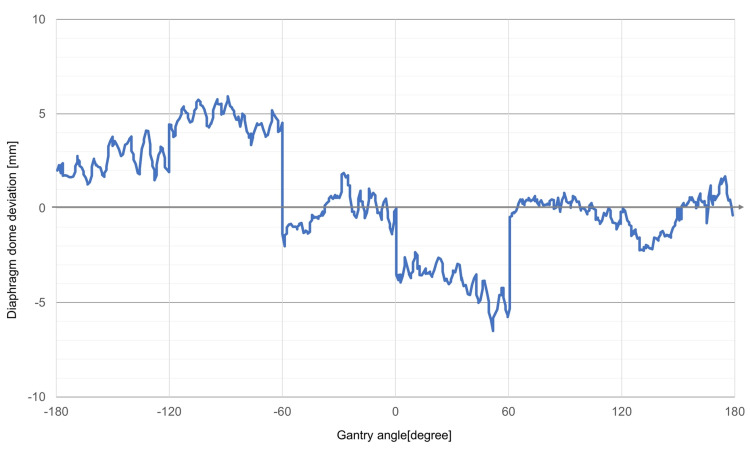
Deviations of diaphragm dome positions The difference of diaphragm dome positions on the digitally reconstructed radiograph and kilovoltage projection images in the superior-inferior direction during breath-hold stereotactic radiotherapy using volumetric modulated arc therapy.

A more accurate displacement was further calculated by quadratic interpolation using the three neighboring data points separated by 1 mm. The mean diaphragm dome deviation was 0.47 mm with a standard deviation of 4.47 mm during the entire arc (Figure 2).

During the whole single-arc radiotherapy from -179 degrees to 179 degrees, 580 sampled data were assessed, and 547 sampled data (91.7 %) were with the deviations less than 5 mm from the reference diaphragm dome position. The mean and standard deviation of each partial arc are represented in Table 1.

**Table 1:** Beam characteristics for each partial arc beam Beam characteristics and diaphragm dome deviations for each partial arc beam of split volumetric-modulated arc therapy. SD: standard deviation.

Partial arc ID	Start gantry angle (deg)	Stop gantry angle (deg)	Monitor unit per fraction (MU)	Duration (second)	Mean of diaphragm dome deviations (mm)	SD of diaphragm dome deviations (mm)
1	-179	-120	283.04	16.9	2.57	0.78
2	-120	-60	310.21	16.9	4.76	0.57
3	-60	0	269.25	17.8	-0.21	0.84
4	0	60	212.67	17.1	-3.87	0.85
5	60	120	168.63	18.0	0.03	0.44
6	120	179	141.6	17.8	-0.47	1.07

The intra-breath-hold standard deviations in the diaphragm position for each partial arc were consistently within 1.1 mm. The primary influencing factor for overall single-arc radiotherapy was the inter-breath-hold variation. The patient successfully completed the treatment regimen without experiencing severe treatment-related toxicities, although mild nausea was reported. Subsequent imaging conducted at the three-month follow-up revealed stable disease with no significant change in tumor size.

## Discussion

Recently, intrafraction breathing motion variation was assessed by other several groups. Stick et al. reported that the maximum intrafractional variation in the implanted fiducial marker position in the superior-inferior direction could be up to 1.0 cm during a single breath-hold [12]. They evaluated the differences in marker position between pre- and post-treatment CBCT scans. Vogel et al. aimed to assess residual motion during deep-inspiration-breath-hold (DIBH) in abdominal SBRT using ultrasound (US) images to monitor the motion of target structures [13]. Approximately 60% of DIBH sessions had a residual motion below 2 mm, which was analyzed by a statistical correlation between US and CBCT measurements.

The main advantage of the present study was the ability to measure intrafraction breathing motion variation during the treatment, not pre- and post-treatment CBCT scans. The findings of this study have highlighted the importance of incorporating real-time imaging techniques into SBRT for HCC. By utilizing kilovoltage projection streaming images, clinicians can assess the actual displacement of the diaphragm dome during treatment, which may differ from the assumptions made during treatment planning based on static DRR images. This dynamic assessment allows for a more accurate estimation of the target position and the potential need for adaptive treatment strategies. Takanaka et al. investigated a technique for multiple breath-hold split-VMAT using implanted fiducial markers and real-time fluoroscopic guidance [14]. Their method, demonstrated in a pancreatic cancer case, shows potential for treating tumors affected by respiration.

One important aspect to consider in future studies is enabling a direct visualization of organ motion and providing immediate feedback to the patient. Nakamura et al. reported that visual feedback significantly improved the reproducibility of wall positions [15]. Yoshitake et al. tested a breath-hold technique with visual feedback using a fiducial marker and a head-mounted display [16]. The participants achieved better reproducibility during expiration breath-holds. This real-time feedback can facilitate adjustments to patient positioning and breath-hold techniques, thereby improving treatment accuracy and reducing potential errors.

Despite the presented promising results, this case report has several limitations. First, it is a single-case report, and the findings should be interpreted with caution until validated in larger patient cohorts. Second, the article focused specifically on liver tumors, and the generalizability of the results to other tumor types or disease stages requires further investigation. Third, our software is currently in development stage, and further studies are required to confirm its robustness and accuracy. Finally, this case report did assess the diaphragm dome, not the position of the tumor location. Tsai et al. analyzed respiratory-induced motion in different liver segments using helical CT [17]. They emphasized the need for individual segment expansion margins in target delineation. Future research should aim to address these limitations and provide more comprehensive evidence on the clinical implications of real-time imaging for intra-breath-hold variation assessment during SBRT.

## Conclusions

To our best knowledge, this case report presents the first case report of intra-breath-hold variation assessment of the diaphragm dome using real-time kilovoltage projection streaming images in reference to DRR images during SBRT for liver tumors. The results have demonstrated the feasibility and potential benefits of real-time imaging in evaluating respiratory motion and optimizing treatment accuracy. Incorporating real-time imaging techniques into SBRT workflows can provide valuable insights into intra-breath-hold variations and guide adaptive treatment strategies that may improve the outcomes of HCC patients undergoing SBRT. Further research is warranted to validate these findings.
